# Effects of Sodium-Glucose Cotransporter 2 Inhibitors on Water and Sodium Metabolism

**DOI:** 10.3389/fphar.2022.800490

**Published:** 2022-02-23

**Authors:** Jun Tang, Lifang Ye, Qiqi Yan, Xin Zhang, Lihong Wang

**Affiliations:** ^1^ The Second Clinical Medical College, Zhejiang Chinese Medical University, Hangzhou, China; ^2^ Department of Cardiovascular Medicine, Zhejiang Provincial People’s Hospital, People’s Hospital of Hangzhou Medical College, Hangzhou, China

**Keywords:** sodium-glucose cotransporter 2 inhibitor, heart failure, diuretic effect, natriuresis, glycosuria

## Abstract

Sodium-glucose cotransporter 2 (SGLT2) inhibitors exert hypoglycemic and diuretic effects by inhibiting the absorption of sodium and glucose from the proximal tubule. Currently available data indicate that SGLT2 inhibitors transiently enhance urinary sodium excretion and urinary volume. When combined with loop diuretics, SGLT2 inhibitors exert a synergistic natriuretic effect. The favorable diuretic profile of SGLT2 inhibitors may confer benefits to volume management in patients with heart failure but this natriuretic effect may not be the dominant mechanism for the superior long-term outcomes observed with these agents in patients with heart failure. The first part of this review explores the causes of transient natriuresis and the diuretic mechanisms of SGLT2 inhibitors. The second part provides an overview of the synergistic effects of combining SGLT2 inhibitors with loop diuretics, and the third part summarizes the mechanisms of cardiovascular protection associated with the diuretic effects of SGLT2 inhibitors.

## Introduction

Sodium-glucose cotransporter 2 (SGLT2) inhibitors are a relatively new class of hypoglycemic drugs that have been shown to improve cardiovascular outcomes in type 2 diabetes mellitus (T2DM) (Zinman et al., 2015; Neal et al., 2017; Wiviott et al., 2019). As shown in the DAPA-HF (McMurray et al., 2019) and EMPEROR-reduced trials (Packer et al., 2020), SGLT2 inhibitors notably reduce the risk of worsening heart failure (HF) or death from cardiovascular causes in patients with HF and a reduced ejection fraction (HFrEF). Among these inhibitors, dapagliflozin significantly reduced the risk of the primary composite endpoint in patients with or without diabetes (McMurray et al., 2019). SGLT2 inhibitors have also been shown to decrease mortality in patients with worsening HF (Bhatt et al., 2021). Recently, the American College of Cardiology (ACC) updated the 2017 ACC Expert Consensus Decision Pathway and supported the addition of SGLT2 inhibitors to HFrEF treatment regimens to improve clinical outcomes (Maddox et al., 2021). Dapagliflozin and empagliflozin are listed as Class IA recommendations in the 2021 ESC guidelines for the diagnosis and treatment of acute and chronic heart failure in HF Patients with reduced ejection fraction (LVEF ≤ 40%) (McDonagh et al., 2021). The potential mechanisms of these benefits in cardiovascular protection are unclear; one possibility is the diuretic effect. However, compared to loop diuretics, SGLT2 inhibitors uniquely prevent sodium and glucose reabsorption in the renal proximal tubule, which result in increased sodium chloride delivery to the macula densa, thereby leading to physiological changes in nervous system function, fluid balance, and energy metabolism that differ from the effects of loop diuretics. This review aims to discuss the effects of SGLT2 inhibitors on water and sodium metabolism.

## Effects of SGLT2 Inhibitors on Sodium Metabolism and Their Diuretic Mechanisms

### Changes in Urinary Sodium Excretion

**FIGURE 1:**
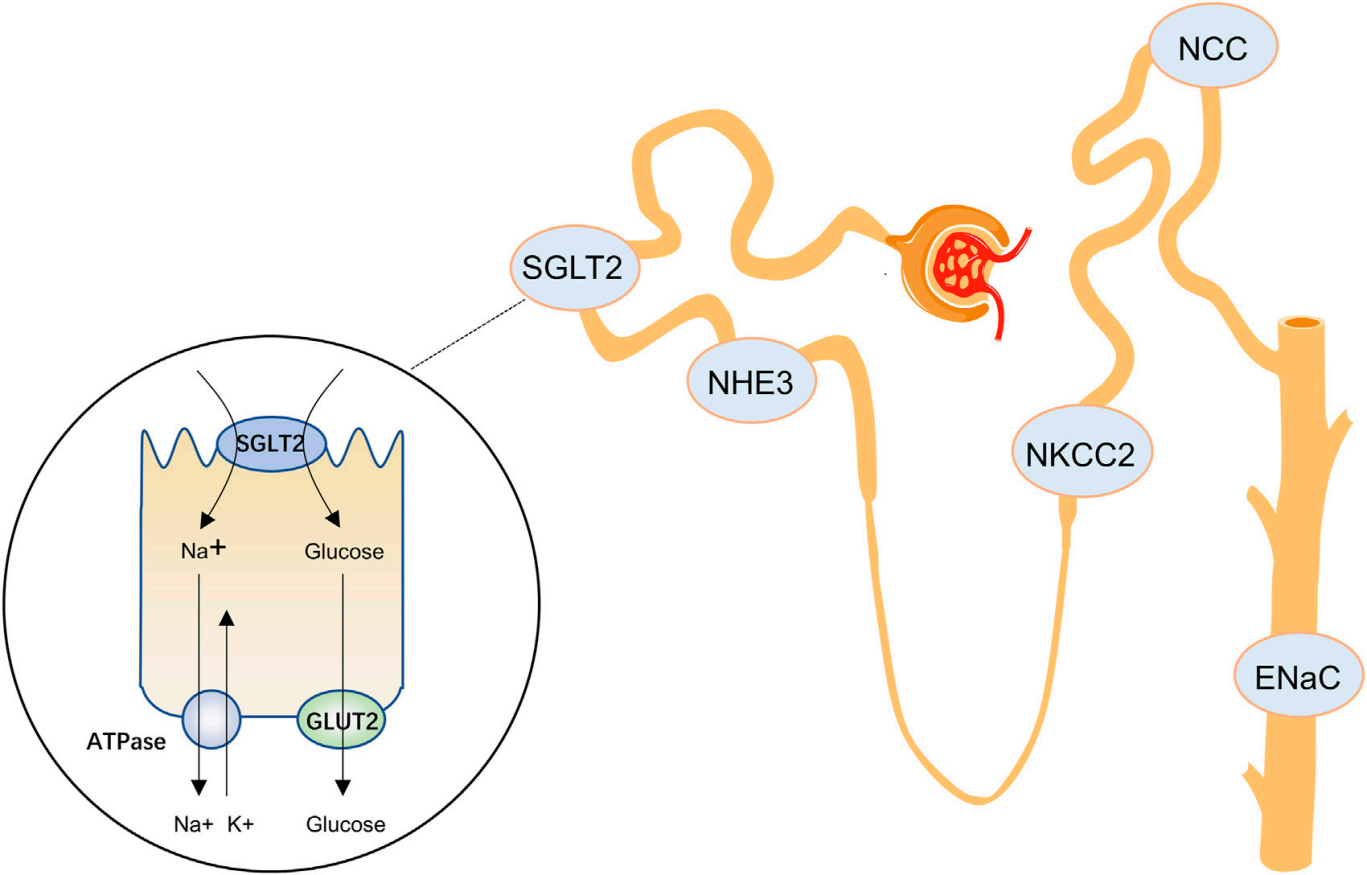
SGLT2 inhibitors and other sodium transporters in the renal tubules. SGLT2, sodium-glucose cotransporter 2; NHE3, Na (+)-H (+) exchanger 3; NKCC2, Na-K-2Cl cotransporter; NCC, Na-Cl cotransporter; ENaC, epithelial sodium channel; GLUT2, glucose transporter 2.

In the kidney, glucose reabsorption is coupled with Na^+^ flux. The Na^+^/K^+^-ATPase in the basolateral membrane of tubular epithelial cells causes the Na^+^ concentration in tubular epithelial cells to be lower than that in the glomerular filtrate. Na^+^ is passively transferred into the cell along the electrochemical gradient, and glucose enters the cell together with Na^+^ at a 1:1 ratio through the SGLT following the Na^+^ concentration gradient, and then passively leaves the cell through the basolateral glucose transporter 2 (GLUT2) (Figure 1). SGLT2 is a low-affinity, high-capacity transporter located in the brush border membrane of the S1 segment of the renal tubules and is almost exclusively expressed in the kidney, accounting for approximately 90% of glucose reabsorption (Kanai et al., 1994; Bakris et al., 2009; Liu et al., 2012). Thus, inhibition of SGLT2 reduces the reabsorption of Na^+^ and glucose in the proximal tubule. SGLT1 is expressed in the S3 segment of the renal tubules and absorbs the remaining 10% of glucose (Liu et al., 2012). Unlike SGLT2, SGLT1 is also widely expressed in the small intestine, where it helps absorb glucose or galactose. Inhibition of SGLT1 may lead to severe diarrhea and dehydration (Wright et al., 2011), so high selectivity for SGLT2 versus SGLT1 is necessary in SGLT2 inhibitors. Among empagliflozin, dapagliflozin, and canagliflozin, which are mainly used in clinical practice, empagliflozin demonstrated higher SGLT2 selectivity over SGLT1 (>2500-fold), whereas canagliflozin showed lower SGLT2 selectivity (155-fold) (Grempler et al., 2012; Kurosaki and Ogasawara, 2013).

In most studies, early treatment with SGLT2 inhibitors resulted in an increase in urinary sodium concentration and urine output. In some studies, these changes in urinary sodium levels were not confirmed (Table 1). A 6-weeks clinical trial in patients with T2DM demonstrated no significant change in the fractional excretion of sodium (FENa) at 6 months compared with placebo (Opingari et al., 2020). Treatment with dapagliflozin in patients with T2DM on a standard diet did not result in significant changes in urine volume or 24-h urinary sodium excretion, although urinary sodium excretion tended to be increased on day 1 (Scholtes et al., 2021). In healthy volunteers, a sharp increase in the FENa during the first 180 min was observed but no significant changes in urinary sodium excretion were noted after 1 month (Zanchi et al., 2020). Experimental data in early diabetic rats showed that urinary sodium and urinary chloride excretion were increased by acute administration of dapagliflozin, but chronic treatment had no significant effect (Thomson et al., 2012). A study of canagliflozin found that urinary sodium excretion rapidly increased within 24 h but returned to baseline after 96 h (Blau et al., 2018). Based on these data from animal studies and clinical trials, the effects of SGLT2 inhibitors on urinary sodium excretion and diuresis appear to be transient.

**TABLE 1:** SGLT2 inhibitors-induced changes in urinary sodium excretion and urinary volume.

	Subjects	Observation period	Food restriction etc.	Urinary sodium excretion	Urine volume	SGLT2 inhibitor	References
Animal experiments	Diabetic Wistar rats	Acute blockage	Standard diet	Increased by ∼2.70 fold	N/A	dapagliflozin	Thomson et al. (2012)
		Chronic blockage		No change			
	SD rats	8 weeks	No	Increased by ∼1.21 fold	Increased by ∼1.60 fold	ipragliflozin	Masuda et al. (2018)
	SDT rats			No change	No change		
	GK rats	8 weeks	No	Increased by ∼1.26 fold	Increased by ∼1.63 fold	ipragliflozin	Masuda et al. (2020)
	SHRcp rats	7 days	Standard diet	Increased by ∼1.24 fold	Increased by ∼2.0 fold	empagliflozin	Kusaka et al. (2016)
		10 weeks		No change	Increased by ∼2.22 fold		
Clinical studies	T2D	18 days	Standard diet	No change	No change	dapagliflozin	Scholtes et al. (2021)
	Healthy volunteer	180 min	No	Increased by ∼1.47 fold	N/A	empagliflozin	Zanchi et al. (2020)
		1 month		NO change			
	T2D	Day 1	No	Increased by ∼1.20 fold	Increased by ∼1.09 fold	canagliflozin	Tanaka et al. (2017)
		7 days		No change	No change		
	T2D with heart failure	3 days	No	No change	mean difference, 535 ml (95% CI, 133–936)	empagliflozin	Mordi et al. (2020)
		6 weeks		No change	mean difference, 545 ml (95% CI, 136–954)		
	Acute heart failure	96 h	No	No change	N/A	empagliflozin	Boorsma et al. (2021)
		30 days		No change			
	T2D and chronic, stable heart failure	3 h	No	Increased by ∼1.71 fold	N/A	empagliflozin	Griffin et al. (2020a)
		14 days		Increased by ∼1.47 fold			
	Healthy subjects	24 h	a fixed diet with ≈110 mmol·d−1 of Na+	Increased by ∼1.34 fold	N/A	dapagliflozin	Wilcox et al. (2018)

SGLT2, sodium-glucose cotransporter 2; T2D, type 2 diabetes; SD, Sprague-Dawley; SDT, spontaneously diabetic Torii; GK, Goto-Kakizaki; SHRcp, metabolic syndrome SHR/NDmcr-cp (+/+); N/A, not available.

Under nondiabetic conditions, SGLT2 is responsible for approximately 5% of the total renal NaCl reabsorption. In the setting of hyperglycaemia in diabetes mellitus, SGLT2 activity accounts for even more NaCl reabsorption as a consequence of increased SGLT2 mRNA expression (Pollock et al., 1991; Vestri et al., 2001). This means that sodium flux increases after SGLT2 inhibition; therefore, increased sodium reabsorption by other nephrons is thought to be the leading cause of transient natriuresis. The most plausible explanation is that sodium absorption by other sodium transporters is increased after SGLT2 inhibition, but currently there is insufficient evidence for this. The Na^+^-H^+^ exchanger 3 (NHE3) is expressed in the proximal tubules. This transporter colocalizes with SGLT2 and mediates Na^+^ reuptake by the renal tubules. Increased expression of NHE1 and NHE3 has been observed in patients with HF (Pessoa et al., 2014; Packer, 2017). Empagliflozin enhanced the renal expression of NHE3 phosphorylated at S605 in diabetic rats (Masuda et al., 2018; Onishi et al., 2020). Whereas phosphorylation of N605 at S605 was associated with reduced NHE3 activity (Rieg et al., 2012), suggesting that SGLT2 inhibitors decrease the activity of NHE3, thereby increasing natriuresis. Reduced NHE3 activity may be one of the reasons why SGLT2 inhibitors prevent the progression of HF (Packer et al., 2017; Borges-Júnior et al., 2021). The Na^+^-K^+^-2Cl^-^ cotransporter (NKCC2) is located in the apical membrane of epithelial cells within the thick ascending limb of the Henle ring and contributes to the reabsorption of approximately 20–25% of the filtered total NaCl load. NKCC2 is a transporter with one of the highest absorption capacities in the kidney (Castrop and Schießl, 2014). It is also the target of the commonly used loop diuretics bumetanide and furosemide (Hannaert et al., 2002). In diabetic rats, NKCC2 protein expression is increased (Kim et al., 2004; Chen et al., 2016). However, dapagliflozin does not affect the diabetes mellitus-induced upregulation of aquaporin (AQP)2 and NKCC2 proteins (Chen et al., 2016). In other studies, inhibition of SGLT2 did not upregulate the expression of NKCC2 (Chung et al., 2019; Ma et al., 2019). Similarly, other sodium transporters, such as the thiazine-sensitive Na^+^-Cl^−^ cotransporter (NCC), also showed no significant change. However, inhibition of SGLT2 increased the mRNA expression levels of Na^+^/phosphate cotransporter (NAPI-2a) and epithelial Na^+^ channel (ENaCα) (Ma et al., 2019).

Another possible reason for increased sodium reabsorption is that SGLT2 inhibitors reduce plasma volume and lower blood pressure, leading to activation of the sympathetic nervous system (SNS) and renin-angiotensin-aldosterone system (RASS).

On the contrary, lowering blood pressure with SGLT2 inhibitors does not increase heart rate (Wan et al., 2018). Previous animal experiments showed that SGLT2 inhibitors even inhibit sympathetic nerve activity (Yoshikawa et al., 2017; Zhang et al., 2019; Herat et al., 2020). For aldosterone, SGLT2 inhibitors have little effect, with only a slight increase in aldosterone at the beginning of treatment (Scholtes et al., 2021).

Ansary et al. (2019) suggested that treatment with SGLT2 inhibitors temporarily activates systemic RAS, but not intrarenal RAS, in patients with T2DM. The candidate mechanism by which SGLT2 inhibitors cause diuresis but rarely cause neurohumoral activation may be that SGLT2 inhibitors inhibit proximal sodium transport and increase salt transport to the macula densa, while loop diuretics directly antagonize NaCl entry into the macula densa, as evidenced by a reduction in glomerular filtration rate after the initiation of SGLT2 inhibitor therapy (Barnett et al., 2014; Ridderstråle et al., 2014; Rosenstock et al., 2014; Wanner et al., 2016). Changes in glomerular filtration rate are thought to be secondary to tubuloglomerular feedback and are a response after the dense macula senses increased salt delivery (Heerspink et al., 2016). Based on the above discussion, the specific location and causes of increased sodium reabsorption are not well understood and need to be further investigated in the future.

### Diuretic Mechanism of SGLT2 Inhibitors

SGLT2 inhibitors simultaneously reduce Na^+^ and glucose reabsorption in the proximal tubule and reduces the osmotic gradient between the distal tubular fluid and the interstitium, resulting in osmotic diuresis. Therefore, the question is whether SGLT2 inhibitors stimulates osmotic diuresis mainly through glycosuria or natriuresis. A study found a slight increase in 24-h urine volume on day 1 in the canagliflozin group compared to the placebo group. Interestingly, the change from baseline in hourly urinary sodium excretion on day 1 showed a time-course profile similar to that of the hourly urine volume (Iijima et al., 2015). Similarly, in another study on canagliflozin, a post hoc multiple regression analysis showed changes in sodium excretion and water intake as factors that affected urine volume changes on day 1 (Tanaka et al., 2017). In 40 patients with T2DM treated with a 50-mg dose of ipragliflozin once daily, changes in 24-h urine volume were closely related to changes in 24-h urine sodium excretion and not significantly related to changes in 24-h urine glucose excretion (Fukuoka et al., 2020). However, in a 6-weeks trial, empagliflozin significantly increased urine volume and urinary glucose excretion, but not urinary sodium excretion (Mordi et al., 2020). Similarly, urinary volume and urinary glucose excretion were increased in patients with acute HF, whereas urinary sodium excretion did not change over 30 days of empagliflozin treatment (Boorsma et al., 2021). Based on these findings in conjunction with the transient natriuretic effects of SGLT2 inhibitors discussed above, we are inclined to hypothesize that since proximal tubule sodium uptake is inhibited and distal compensation mechanisms of the tubule are not activated, both natriuresis and glycosuria are present, as shown in the rat nephron model (Weinstein, 2015). In this time frame, natriuretic diuresis may play a dominant role in the treatment with SGLT2 inhibitors. Over time, a new sodium balance is gradually established. Although sodium uptake in the proximal tubule is still inhibited, the effect of natriuretic diuresis slowly diminishes due to increased sodium reabsorption in the distal tubule and a part of neurohumoral influences. In a mouse model of metabolic syndrome treated with ipragliflozin, urine volume and urinary glucose excretion rate continued to be higher than those in the control group after long-term treatment (10 weeks). Unlike the short-term (7 days) treatment effect, there were no significant differences in urinary sodium excretion and 24-h sodium balance until the end of the treatment, although there was still a significant interaction regarding 24-h water balance. This animal study supports our hypothesis regarding the duration of SGLT2 inhibitor therapies (Kusaka et al., 2016). The glucosuria persists during treatment with SGLT2 inhibitors, but not urine volume, which may be related to the generation and accumulation of urea by changing energy metabolism under dehydration stress. In the kidney, urea is the most potent organic osmolyte for urine concentration, and additional urea permeates are synthesized to increase the concentration of renal interstitial permeates by enhancing the hepatic urea cycle to counteract the osmotic diuresis induced by SGLT2 inhibitors (Kitada et al., 2017; Kitada et al., 2021; Marton et al., 2021). Previous studies showed that dapagliflozin does increase urea transporter U1-AT levels in rats (Chen et al., 2016). In the liver, the production of a urea molecule requires two alanine transamination to produce nitrogen transfer and three ATP molecules; the process requires energy and protein consumption, which comes from food intake when nutrients are sufficient, or through the liver glycogen and endogenous protein reservoirs from skeletal muscle in the absence of adequate food (Marton et al., 2021). At the same time, antidiuretic hormone, also known as vasopressin, plays an important role in renal water preservation. It not only promotes the reabsorption of free water in renal tubules, but also promotes the transamination of amino acids and indirectly increases the absorption of urea by the kidney. Vasopressin acts on the V2 receptor (V2R) in the collecting duct and is mediated *via* water transport across aquaporin 2 (AQP2) channels (Knepper et al., 2015). In diabetic rat kidneys, chronic administration of empagliflozin was shown to decrease total AQP2 expression and increases AQP2 phosphorylation at S261 (Chung et al., 2019). Masuda et al. (2020) found that ipragliflozin increases the protein expression levels of AQP2 phosphorylated at Ser269 and vasopressin V2 receptor at renal cell membranes. Ultimately, the SGLT2-induced increase in urine volume does not persist under the combined effect of an increase in urea solutes and vasopressin-mediated water reabsorption.

## Synergism Between SGLT2 Inhibitors and Loop Diuretics

Diuretics are widely used in patients with HF, and 93% of patients in the DAPA-HF study received diuretics at baseline. In the DAPA-HF study (McMurray et al., 2019), dapagliflozin reduced the risk of death from cardiovascular causes in patients with HFrEF, irrespective of the presence or absence of diabetes mellitus. Therefore, as a potential HF therapy, SGLT2 inhibitors may be frequently used with loop diuretics.

The combination of SGLT2 inhibitors with loop diuretics exerts synergistic effects (Heise et al., 2016; Wilcox et al., 2018; Griffin et al., 2020a). In a randomized, double-blind, placebo-controlled trial, the enhancement of FENa during coadministration of SGLT2 inhibitors and loop diuretics was >4-fold greater than that observed during monotherapy (Griffin et al., 2020a). However, in another study, the initial Na^+^ excretion following bumetanide plus dapagliflozin administration was not greater than that after bumetanide treatment alone (Wilcox et al., 2018), indicating no first‐dose synergy between this SGLT2 inhibitor and the loop diuretic.

SGLT2 inhibitors increase urine volume and net fluid balance when combined with loop diuretics (Damman et al., 2020). The therapeutic aim in acute decompensated HF is to relieve congestion (Gheorghiade et al., 2006). However, during the use of loop diuretics, diuretic resistance often occurs. Patients receive a large number of diuretics intravenously, and the intravascular volume does not improve (Testani et al., 2013; Testani et al., 2014), which is associated with the induction of neurohormonal system responses and the rapid deployment of sodium-sparing mechanisms (Felker and Mentz, 2012; Shah et al., 2017). In this setting, thiazide diuretics are often recommended as first-line diuretic adjuvants (Yancy et al., 2013). However, these drugs are associated with the development of hypokalaemia, hyponatremia, and worsening renal function (Jentzer et al., 2010; Brisco-Bacik et al., 2018). SGLT2 inhibitors have good safety and tolerability, with few reports regarding a decline in renal function, hypokalaemia, or hyponatremia (Zinman et al., 2015; McMurray et al., 2019; Damman et al., 2020; Mordi et al., 2020; Boorsma et al., 2021). In addition, compared to conventional diuretics, SGLT2 inhibitors reduce the tissue fluid volume more than the blood volume (Hallow et al., 2018; Ohara et al., 2019), which may help to alleviate congestion without excessively reducing intravascular filling. Therefore, some researchers believe that SGLT2 inhibitors may be a more advantageous diuretic adjuvant than thiazides. Some clinical trials reported that patients treated with SGLT2 inhibitors require reduction of loop diuretics dose (Kambara et al., 2019; Singh et al., 2020). After cessation of the SGLT2 inhibitor, congestion increased, requiring additional loop diuretics or even hospitalisation for HF (Mordi et al., 2020). However, in a post hoc analysis of DAPA-HF data including 4,616 patients, most patients showed no change in diuretic dose during the entire follow-up period (Jackson et al., 2020). Interestingly, despite failing to reduce the dose of background diuretics, SGLT2 inhibitors appear to diminish the need for intensification of diuretic therapy. In a double-blind controlled study, the empagliflozin effect to prevent the intensification of diuretic therapy was twice as great as the effect of this drug to promote a dose reduction of diuretics (Packer et al., 2021a). Regarding patients with diuretic resistance, a retrospective study showed weight loss and improved diuretic efficiency in the majority of patients with diuretic-resistant acute decompensated HF who received SGLT-2 inhibitors and loop diuretics (Griffin et al., 2020b). However, the initial loop diuretic doses in this study were substantially high, and the study lacked a randomization group. The EMPEROR-Reduced trial (Packer et al., 2021b) demonstrated that empagliflozin reduces the risk of the composite of cardiovascular death or hospitalisation for HF in patients with or without volume overload compared to placebo. The magnitude of these benefits is not more pronounced in patients with recent volume overload, although these patients are more prone to fluid retention. To date, little data exist on the coadministration of SGLT2 inhibitors and loop diuretics. Whether SGLT2 inhibitors can be an alternative to thiazides remains to be further studied; however, patients with HF benefit from treatment with SGLT2 inhibitors.

## Mechanisms of Cardiovascular Protection Associated With Diuretic Effects

**FIGURE 2:**
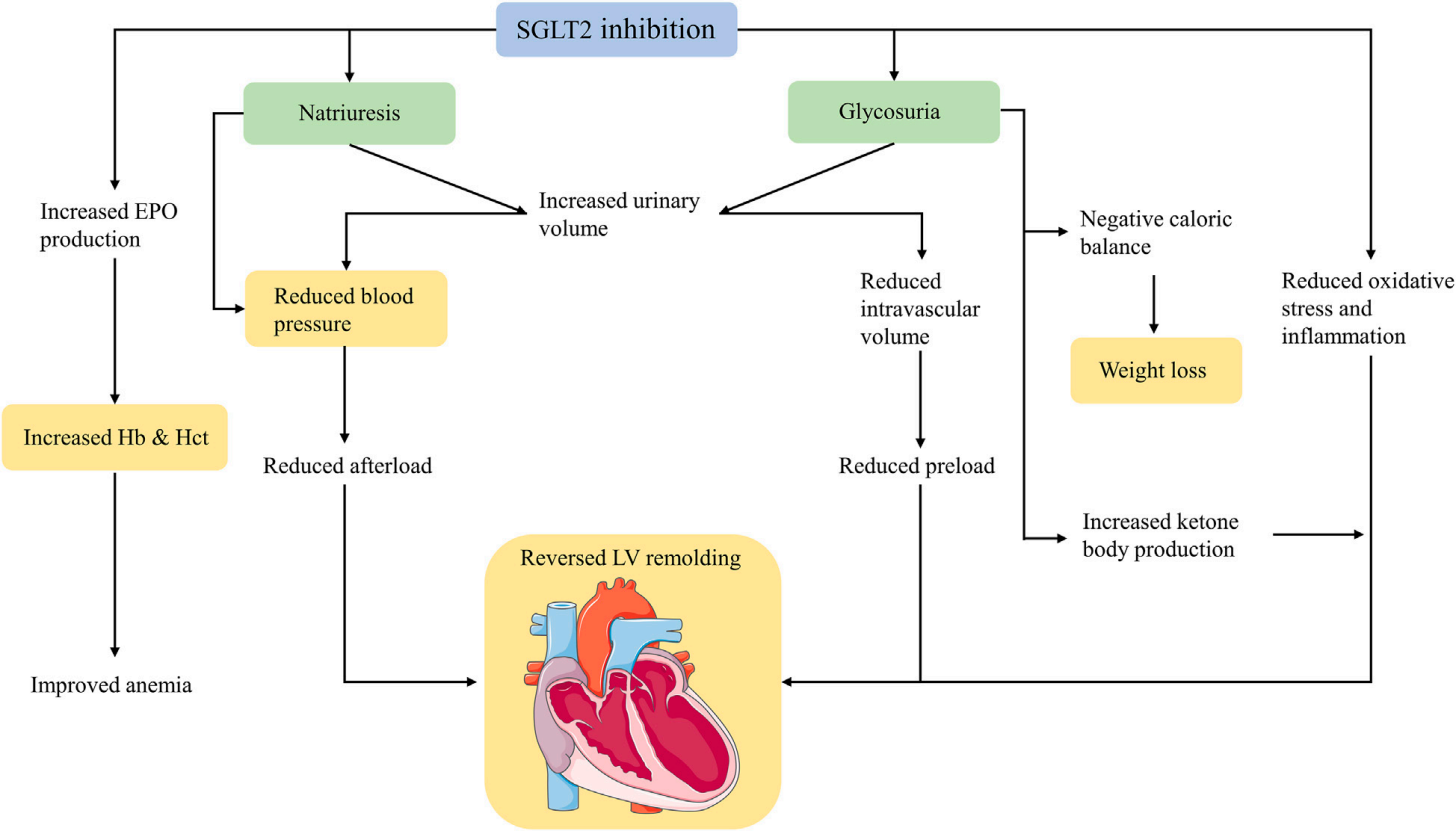
Mechanisms of cardiovascular protection of SGLT2 inhibitors mentioned in this article. SGLT2, sodium-glucose cotransporter 2; EPO, erythropoietin; Hb, haemoglobin; Hct, haematocrit; LV, left ventricular.

The EMPA-REG OUTCOME trial (Zinman et al., 2015), which evaluated the effects of SGLT2 inhibitors in patients with increased cardiovascular risk, showed significant reductions in all-cause mortality, HF hospitalization, and cardiovascular mortality. This finding was unexpected. Since the EMPA-REG OUTCOME trial, an increasing number of hypotheses have been proposed, but no consensus has been reached on the mechanism of cardiovascular protection. SGLT2 inhibitors have a unique diuretic mechanism thought to be one of the reasons for cardiovascular benefits in patients with HF; thus, we summarise the following cardiovascular protective mechanisms associated with diuresis caused by SGLT2 inhibitors (Figure 2).

### Weight Loss

In various studies, patients treated with SGLT2 inhibitors reported weight loss of approximately 1–3 kg. This effect was observed from the first week of treatment, continued to decrease slowly over the following months, and then remained unchanged (Bolinder et al., 2012). The early weight loss was primarily due to rapid fluid loss resulting from natriuresis and glucosuria (Iemitsu et al., 2016; Griffin et al., 2020a). Simultaneously, SGLT2 inhibitors induce glucose excretion, resulting in an energy loss of 200 kcal/day or more, which is responsible for persistent weight loss even after fluid balance. Patients receiving SGLT2 inhibitors have a compensatory increase in glucose intake that counteracts the negative energy balance caused by glycosuria (Horie et al., 2018), and the weight loss is mainly due to a reduction in fat deposition. The study found that after treatment with SGLT2 inhibitors, visceral adipose tissue and subcutaneous adipose tissue were significantly reduced, without affecting muscle mass (Bolinder et al., 2012; Sugiyama et al., 2018). The precise contribution of SGLT2 inhibitor-mediated weight loss to cardiovascular disease has not been elucidated. However, in a post hoc analysis of the randomized clinical Look AHEAD trial (Gregg et al., 2016), diabetic patients who lost at least 10% of their body weight had a 21% reduction in the risk of major cardiovascular outcomes, although the per cent change in body weight with SGLT2 inhibitors is generally lower than that observed in the Look AHEAD post hoc cohort.

### Blood Pressure Decreases

Meta-analyses have consistently demonstrated that SGLT2 inhibitors reduce both systolic and diastolic blood pressure (BP) in both office and ambulatory measurements. A meta-analysis of 27 randomised controlled trials in patients with T2DM found that SGLT2 inhibitors reduced the in-office-measured systolic/diastolic BP by 4.0/1.6 mmHg (Baker et al., 2014). In another meta-analysis of 2,098 participants from six randomised controlled trials, treatment with SGLT2 inhibitors produced a 3.76/1.83 mmHg decline when measured by 24-h ambulatory systolic/diastolic BP monitoring (Georgianos and Agarwal, 2019). Moreover, reductions in daytime pressure were greater than those achieved overnight (Kario et al., 2018; Georgianos and Agarwal, 2019). Regarding the reasons why SGLT2 inhibitors lower BP, theoretical considerations and evidence support the effects of natriuresis and osmotic diuresis. In T2DM patients with preserved renal function, daily administration of a placebo, 10 mg dapagliflozin, or 25 mg hydrochlorothiazide decreased after 12 weeks the ambulatory BP by 0.9, 3.3, and 6.6 mmHg, respectively. The body weight declined in both dapagliflozin and hydrochlorothiazide groups, and the plasma renin and aldosterone levels increased (Lambers Heerspink et al., 2013). In this study, body weight can be seen as a surrogate measure of salt and water loss, whereas the increases in plasma renin and aldosterone reflect a compensatory response to the reduced circulating blood volume. It is well known that natriuresis and osmotic diuresis are based on intact renal function; however, in patients with advanced diabetic nephropathy, the degree of BP reduction is similar to that in patients with preserved renal function (Cherney et al., 2018). Moreover, SGLT2 inhibitors rarely cause diuresis-related electrolyte abnormalities, such as hypokalaemia, hypomagnesemia, and hyperuricemia, suggesting that SGLT2 inhibitors lower BP by other methods in addition to “diuresis,” which may be secondary to reduced arterial stiffness and improved endothelial function. Arteriosclerosis is a risk factor for cardiovascular events and death (Vlachopoulos et al., 2010), and pulse wave velocity (PWV) is a major parameter of arterial stiffness. Empagliflozin significantly reduced aortic PWV in patients with uncomplicated type 1 diabetes mellitus (Cherney et al., 2014). In a post hoc analysis, canagliflozin improved markers of arterial stiffness (replacing PWV with pulse pressure) and reduced myocardial oxygen consumption (evaluated as the product of heart rate and systolic pressure) in patients with T2DM (Pfeifer et al., 2017). Endothelial function is also a factor that affects BP. Endothelial dysfunction can lead to disturbances in the production and utilisation of nitric oxide, contributing to the development of hypertension. In some studies, SGLT2 inhibitors have been observed to improve endothelial function, reduce vascular resistance, and induce vasodilation (Chilton et al., 2015; Solini et al., 2017; Li et al., 2018). These beneficial effects may be mediated *via* activation of voltage-gated K^+^ channels and protein kinase G (Li et al., 2018).

### Haematocrit and Haemoglobin Increases

SGLT2 inhibitors increase haematocrit levels and can even improve anaemia in patients with HF. In a post hoc analysis of DAPA-HF, dapagliflozin corrected anaemia more often than placebo and improved outcomes regardless of the baseline anaemia status (Docherty et al., 2021). Initially, these observations were attributed solely to haemoconcentration due to the diuretic effect of dapagliflozin. We now know that although the increased urine output caused by SGLT2 inhibitors returns to baseline after a few weeks, the haematocrit continues to rise substantially afterwards (Sano and Goto, 2019). Moreover, the assumption that diuresis increases haematocrit has little support from studies in patients with HF given the usual oral doses of conventional diuretics. In studies using conventional oral diuretics, no increase in haematocrit was found (Schuster et al., 1984). SGLT2 inhibitors are more likely to remove interstitial fluid than intravascular fluid; (Ohara et al., 2019) thus, the mechanism driving this effect on the haematocrit cannot be explained by diuresis alone. Evidence suggests that SGLT2 inhibition induces erythropoiesis, and in patients with T2DM, erythropoiesis levels were significantly increased after 1 month of treatment with empagliflozin (Mazer et al., 2020). A 12-weeks study showed that canagliflozin increased erythropoietin concentrations by 38% at 2–4 weeks (Maruyama et al., 2019). It has also been reported that dapagliflozin stimulates erythropoiesis by inhibiting hepcidin (Ghanim et al., 2020). It has been found that increased erythropoietin production by SGLT2 inhibitors is associated with decreased serum ferritin levels (Mazer et al., 2020), suggesting that SGLT2 inhibitors may increase iron utilisation. Notably, erythropoiesis-stimulating agents have failed to improve the clinical outcomes of HF. In the RED-HF trial (Swedberg et al., 2013), treatment with the erythropoietic agent darbepoetin alfa did not reduce mortality or morbidity compared to placebo. However, the physiological changes caused by increases in endogenous erythropoietin levels may be quite distinct from those following the administration of high-dose erythropoietin-inducing drugs; therefore, the possibility of erythropoietin-mediated cardiovascular protection caused by SGLT2 inhibitors cannot be ruled out.

### Left Ventricular Volume and Mass Reduction

To date, the majority of cardiac magnetic resonance imaging studies assessing the effects of SGLT2 inhibitors on cardiac structure and function in patients with T2DM showed a reduction in left ventricular (LV) volume and mass and an improvement in indices of diastolic function, but little change in LV ejection fraction (Matsutani et al., 2018; Verma et al., 2019; Hwang et al., 2020; Lee et al., 2021). In nondiabetic patients with HFrEF, significant reductions in LV volume and left ventricular mass were also observed after 6 months of empagliflozin treatment (Santos-Gallego et al., 2021). The DAPA-LVH trial (Brown et al., 2020) demonstrated that dapagliflozin can initiate reverse remodelling and changes in the LV structure. In a mathematical model of natriuretic/diuretic effects on cardiac haemodynamics, SGLT2 inhibitors reduced blood volume and interstitial fluid volume compared to placebo, resulting in decreased LV end-diastolic volume and pressure (Yu et al., 2021). This suggests that the effects of SGLT2 inhibitors on the improvement of ventricular structure may be associated with haemodynamic alterations brought about by natriuresis/diuresis. SGLT2 inhibitors may cause a reduction in LV afterload by lowering BP and preload by inducing diuresis; the consequent reduction in LV stretch may lead to a reduction in LV volumes. Brain natriuretic peptide (NT-proBNP) is a surrogate marker of the degree of stress in the LV wall (Maeda et al., 1998). The reduction of NT-proBNP levels by SGLT2 inhibitors appears mainly after several weeks of treatment (Packer et al., 2021a), suggesting that SGLT2 inhibitors reverse ventricular remodelling not only through haemodynamic changes.

Under conditions of stress, such as T2DM and HF, the substrates utilized by cardiomyocytes shift from free fatty acids (FFA) to glucose. Although the oxidation of glucose is more oxygen efficient, less energy is produced, and together with decreased mitochondrial oxidative metabolic capacity in failing hearts, leading to cardiac energy deprivation. Ketone bodies are described as super fuels because they produce more energy with less oxygen compare with FFA and glucose (Mudaliar et al., 2016). Interestingly, SGLT2 inhibitors are precisely able to produce energy conservation effects similar to “fasting” with increased levels of circulating ketones in serum, as seen in T2DM (Polidori et al., 2018). In animal studies, empagliflozin increased the levels of circulating ketone bodies, as well as the myocardial expression of a ketone body transporter and two critical ketogenic enzymes (Yurista et al., 2019). In nondiabetic pigs, empagliflozin has also been shown to switch myocardial fuel utilisation away from glucose toward ketone bodies (Santos-Gallego et al., 2019). A hypothesis (Ferrannini et al., 2016) of elevated levels of β-hydroxybutyrate (one of the ketone bodies) offered significant cardioprotection has been suggested and it has been evidenced that chronic infusion of β-hydroxybutyrate could improve canine cardiac function and remodelling dysfunction heart (Horton et al., 2019). Clinically, increased cardiac output and EF have been observed with infusion of β-hydroxybutyrate in patients with HF (Nielsen et al., 2019). Although there is still disagreement on whether SGLT2 inhibitors can enhanced myocardial work efficiency (Ho et al., 2019; Horton et al., 2019; Santos-Gallego et al., 2019), it is certain that SGLT2 inhibitors can supply the extra fuel and change the state of “energy hunger” in failing heart.

Inflammation is an important factor contributing to the progression of HF. Treatment with SGLT2 inhibitors has been evidenced reduce the levels of inflammatory factors including tumor necrosis factor (TNF) -α, interleukin (IL) -6, and C-reactive protein (Han et al., 2017; Garvey et al., 2018; Zhang et al., 2019). At present how SGLT2 inhibitors attenuate inflammation is unknown, may be mediated by multiple factors, such as activated M2 macrophages (Xu et al., 2017), increased levels of ketone bodies (Youm et al., 2015), and decreased sympathetic tension in the aorta (Zhang et al., 2019). Moreover, SGLT2 inhibitors decrease cardiomyocyte hypertrophy, suppress myocardial oxidative stress, and attenuate myocardial fibrosis (Habibi et al., 2017; Lee et al., 2017; Li et al., 2019; Yurista et al., 2019). These effects may be related to the improvement of myocardial expression of SGK1 and ENaC (Habibi et al., 2017), inhibition of the transforming growth factor β/Smad pathway (Li et al., 2019), and regulation of macrophage polarisation *via* STAT3 signalling (Lee et al., 2017).

The unique diuretic mechanism of SGLT2 inhibitors that distinguishes them from classical diuretics is undoubtedly one of the reasons why they have attracted much attention. In the early stages of treatment, SGLT2 inhibitors provide benefit through changes in haemodynamics and natriuresis, as shown by decreases in body weight, reduction in blood pressure, and increases in haematocrit. However, this effect is transient, with changes in weight, BP, and haematocrit remaining after the end of the diuretic effect. Thus, the effects of SGLT2 inhibitors on these physiological parameters appear to be explained by the non-diuretic effects of these agents. Additionally, SGLT2 inhibitors have minimal effects on the average dose of prescribed diuretics (Jackson et al., 2020). Packer et al. (2021b) conclude that diuresis induced by SGLT2 inhibitors does not play a dominant role in physiological changes and clinical benefits in the course of HFrEF. Therefore, future studies should focus more on SGLT2 inhibitor effects other than diuresis and plasma volume reduction. Currently, cardiovascular beneficial mechanisms such as endogenous ketone production, myocardial calcium handling, alterations in epicardial fat thickness and properties, and modulation of sympathetic activity by SGLT2 inhibitors are being discovered, and more mechanisms may await exploration in the future.

## Conclusion

The reduction in the risk of the primary endpoint of HF observed in a large clinical trial of SGLT2 inhibitors is unexpected and impressive. SGLT2 inhibitors alter the metabolism of water, sodium, and energy, eliciting a range of downstream responses that underlie the cardiovascular benefits, although urinary sodium excretion and diuresis are not always sustained. At present, changes in plasma volume and hemodynamic caused by diuresis may not be the main contributing mechanism to the great clinical benefit of SGLT2 inhibitors, while changes in energy metabolism caused by glycosuria need to be further studied.
